# Supplementary material for: Layered polymer-perovskite composite membranes for ultraflexible fatigue-tolerant optoelectronics
Source: Nat Commun. 2025 Jul 1;16:5584. doi: 10.1038/s41467-025-60705-5 (PMC12215640; doi:10.1038/s41467-025-60705-5)
Supplement: Supplementary file 1 — Supplementary Information [file 41467_2025_60705_MOESM1_ESM.pdf]

Supplementary information

Layered polymer-perovskite composite membranes for ultraflexible fatigue-tolerant optoelectronics

Yalu Li[1], Can Zou[1], Da Liu[1], Qing Li[1], Yan Zhu[1], Miaoyu Lin[1], Sihan Zeng[1], Zhanpeng Wei[1], Xinyi Liu[1], Yichu Zheng[2], Yu Peng[1], Yu Hou[1]*, Hua Gui Yang[1]*, Shuang Yang[1]*

[1]Key Laboratory for Ultrafine Materials of Ministry of Education, Shanghai Engineering Research Center of Hierarchical Nanomaterials, School of Materials Science and Engineering, East China University of Science and Technology, Shanghai 200237, China

[2]School of Mechatronic Engineering and Automation, Shanghai University, 99 Shangda Road, 200444, Shanghai, China

*Correspondence: yhou@ecust.edu.cn (Y.H.), hgyang@ecust.edu.cn (H.G.Y.), syang@ecust.edu.cn (S.Y.)

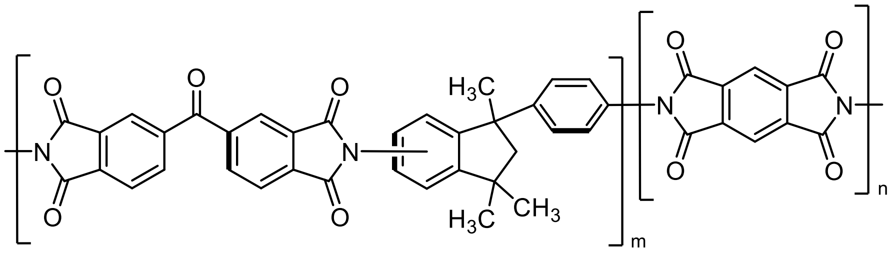

Supplementary Fig. 1 | Chemical structure of polyimide used in this work.

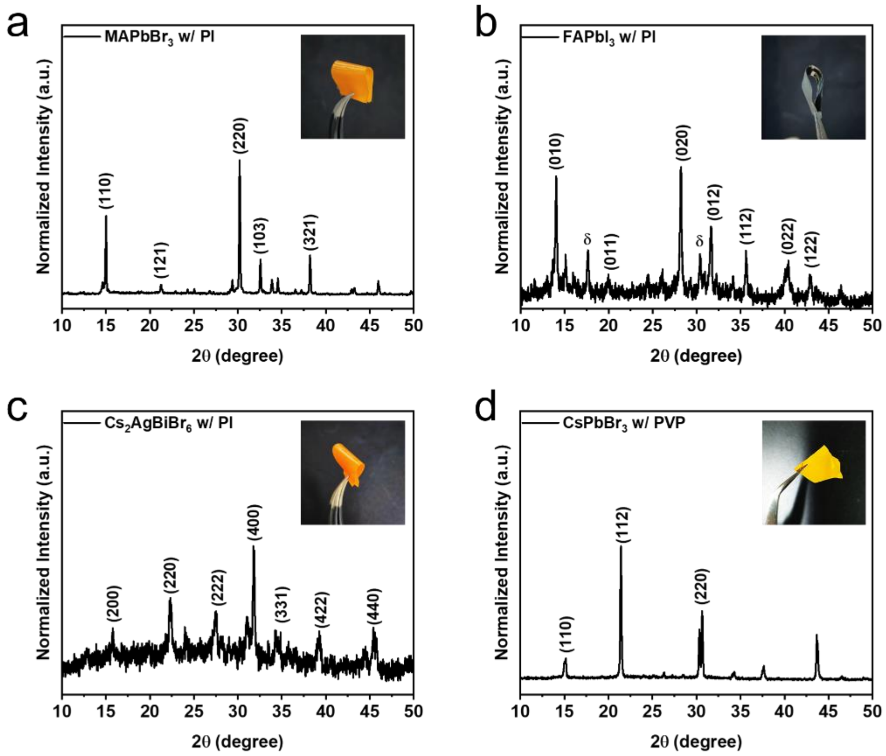

Supplementary Fig. 2 | XRD patterns of composite membranes with different compositions: (**a**) $MAPbBr_3$ w/ PI, (**b**) $FAPbI_3$ w/ PI, (**c**) $Cs_2AgBiBr_6$ w/ PI and (**d**) $CsPbBr_3$ w/ PVP. The insets are photographs of composite membranes.

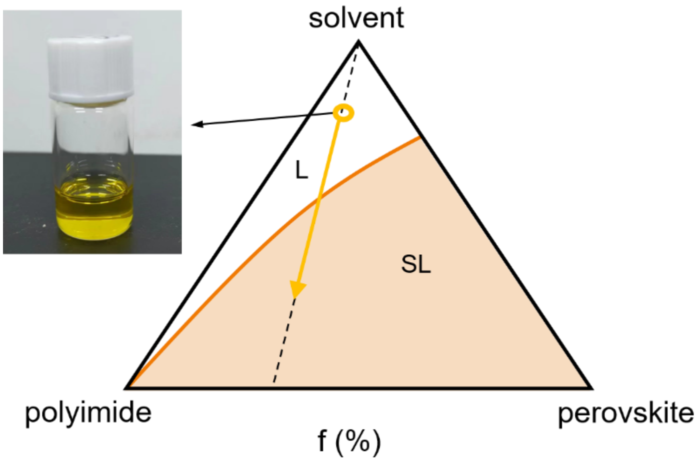

Supplementary Fig. 3 | Schematic phase diagram of the ternary perovskite/polyimide/solvent system at constant temperature and pressure. The perovskite solubility in the polyimide/solvent mixtures is indicated by orange solid line. The ternary mixtures system is homogeneous in the white regions (L) showing like the picture and crystallization of perovskite occurs in the orange regions (SL). The yellow arrow indicates the drying process of the ternary mixtures solution at a certain mass fraction of perovskite (f).

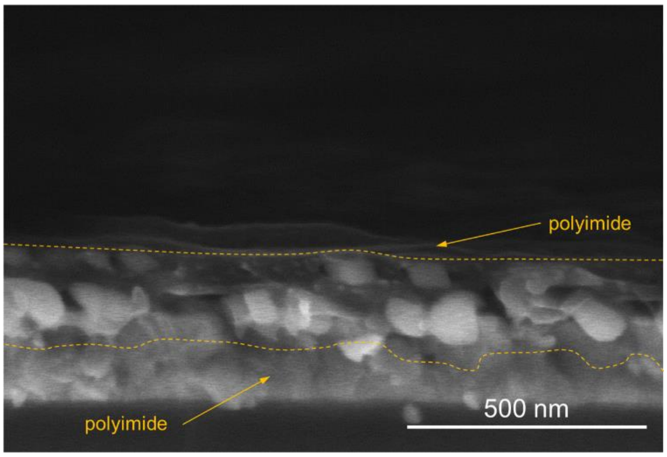

Supplementary Fig. 4 | Cross-section SEM image of the composite membrane processed by single cycle of spray-coating. Scale bar is 500 nm.

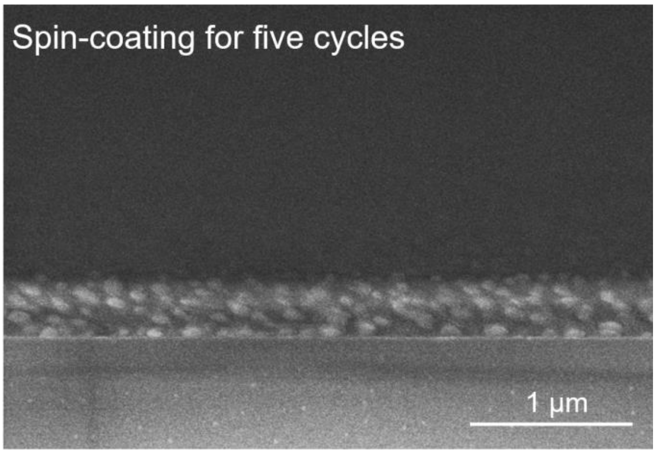

Supplementary Fig. 5 | Cross-section SEM image of the composite membrane processed by spin-coating for five cycles. Scale bar is 1 μm.

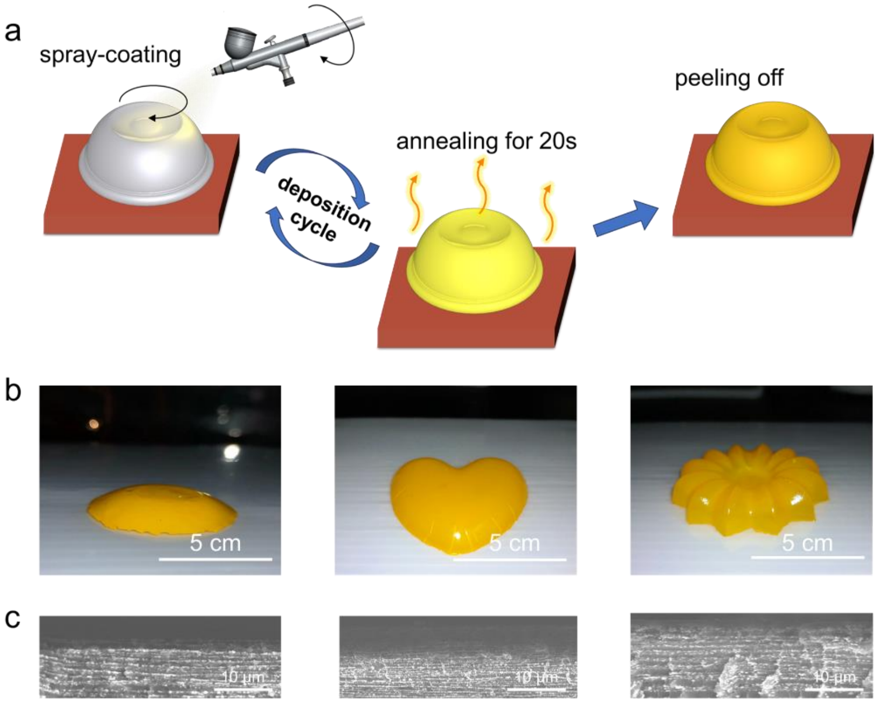

Supplementary Fig. 6 | a, Schematic diagram of spray-coating process on the aluminum alloy substrate with irregular shape. **b**, Photographs of composite membranes with irregular shapes. The membranes can be easily deteched form aluminum alloy substrates due to the weak interfacial interaction. Scale bars are 5 cm. **c**, Cross-sectional SEM images of the membranes. Scale bars are 10 μm.

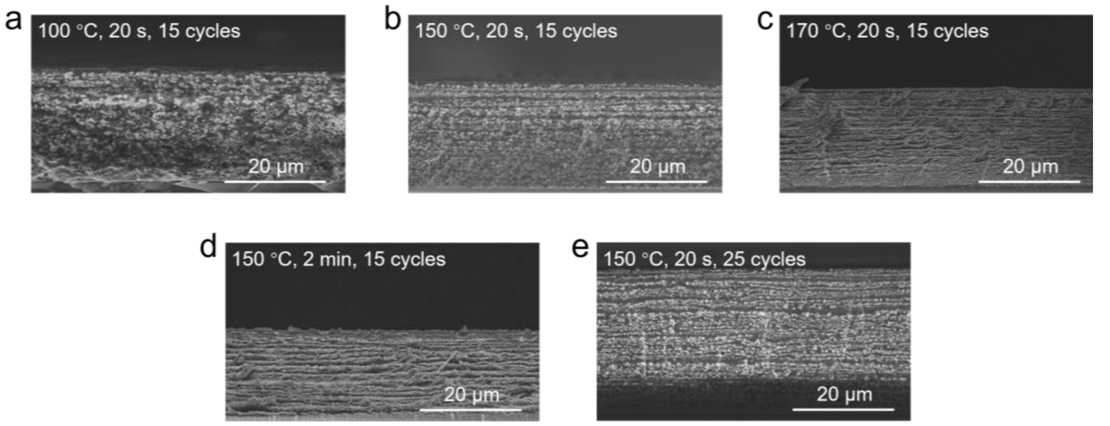

Supplementary Fig. 7 | Cross-sectional SEM images of composite membranes. Spray for 15 cycles and anneal for 20 s at different anneal temperatures: (**a**) 100 °C, (**b**) 150 °C and (**c**) 170 °C. **d**, Spray for 15 cycles and anneal for 2 min at 150 °C. **e**, Spray for 25 cycles and anneal at 150 °C for 20 s. Scale bars are 20 μm.

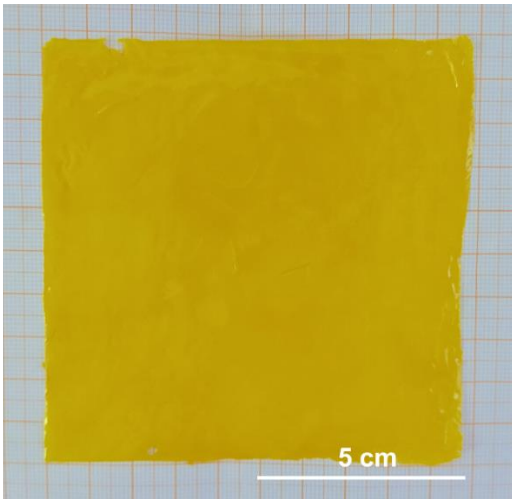

Supplementary Fig. 8 | Photograph of a large-area composite membrane (10 × 10 cm^2) peeled from glass substrate. Scale bar is 5 cm.

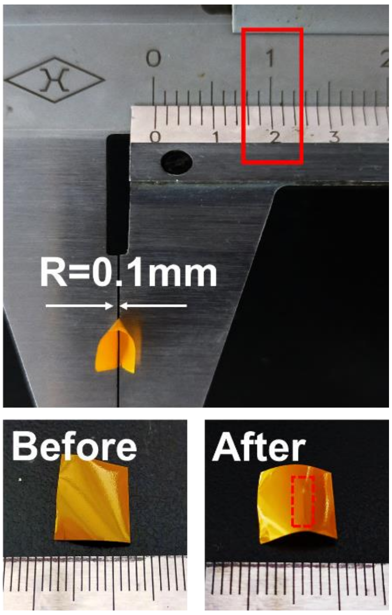

Supplementary Fig. 9 | Bending experiment of the composite membrane to a bending radius of 0.1 mm. The membrane was almost fully recovered after the bending test, showing ultra-flexibility.

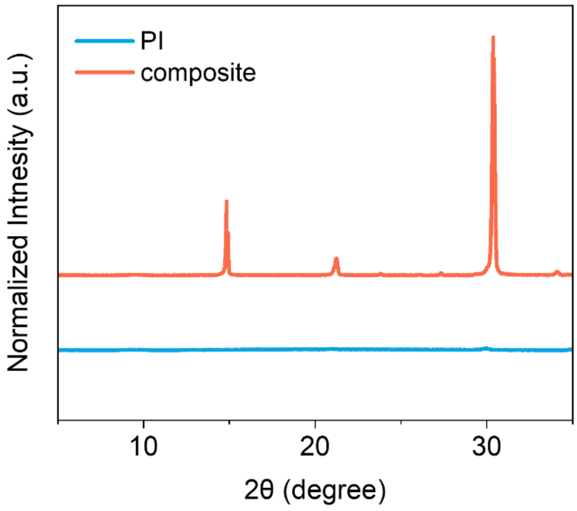

Supplementary Fig. 10 | XRD patterns of pure PI membranes and composite membranes.

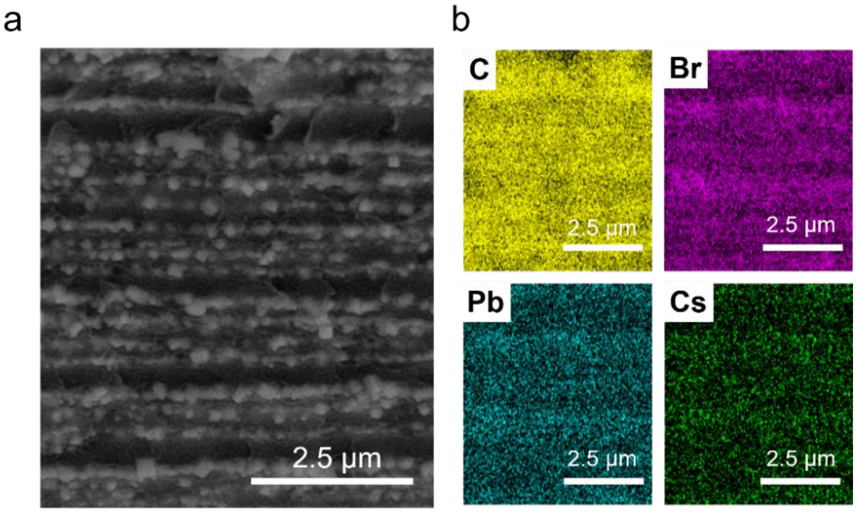

Supplementary Fig. 11 | (a) Cross-sectional SEM image and (**b**) EDX analysis showing the elemental distribution of C, Cs, Pb, and Br in the composite membrane. Scale bars are 2.5 μm.

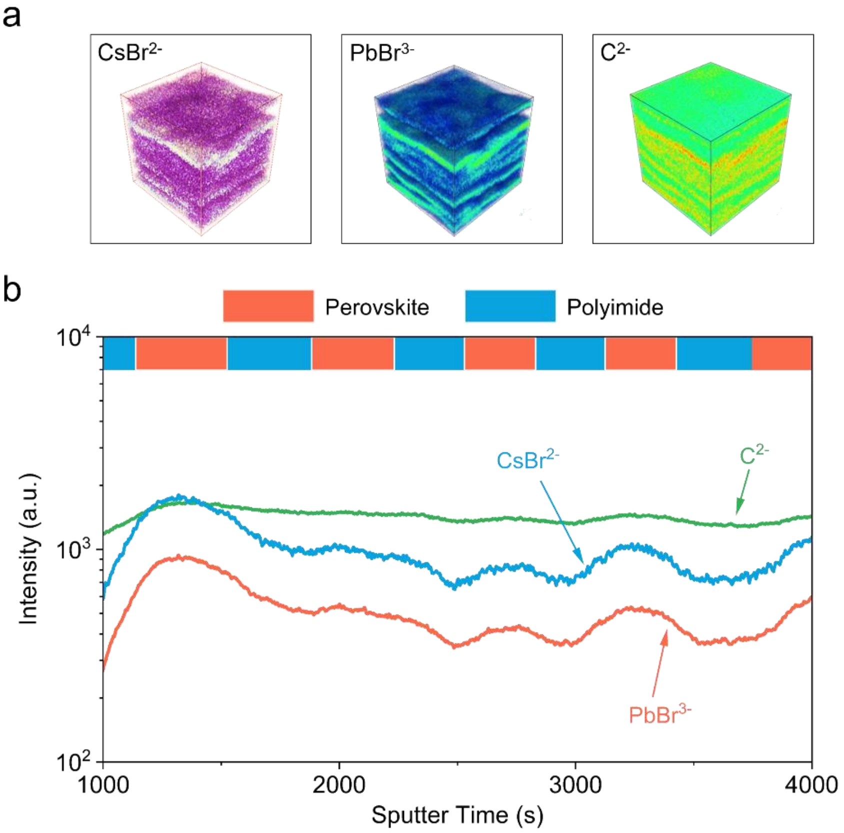

Supplementary Fig. 12 | (a) Reconstructed TOF-SIMS 3D images and (**b**) depth profiles of $CsBr^{2-}$, $PbBr^{3-}$, and C^{2-} signals of the composite membrane. The x-y dimensions of the analyzed area of the membrane were 100 μm × 100 μm.

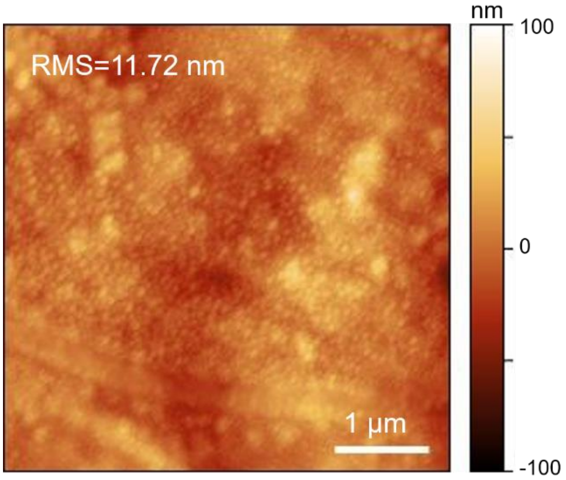

Supplementary Fig. 13 | AFM height image of the composite membrane. The root mean square roughness was measured to be 11.72 nm. Scale bar is 1 µm.

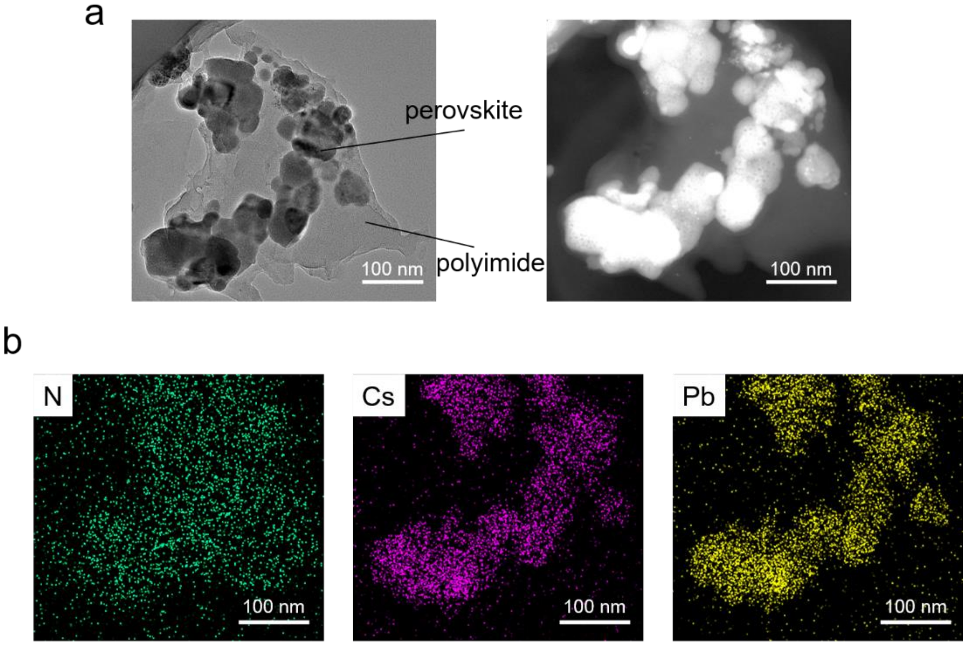

Supplementary Fig. 14 | (**a**) TEM images and (**b**) EDX analysis of a fragment of the composite membrane. Perovskite crystals were loaded on the PI sheet with a biphase heterostucture. Scale bars are 100 nm.

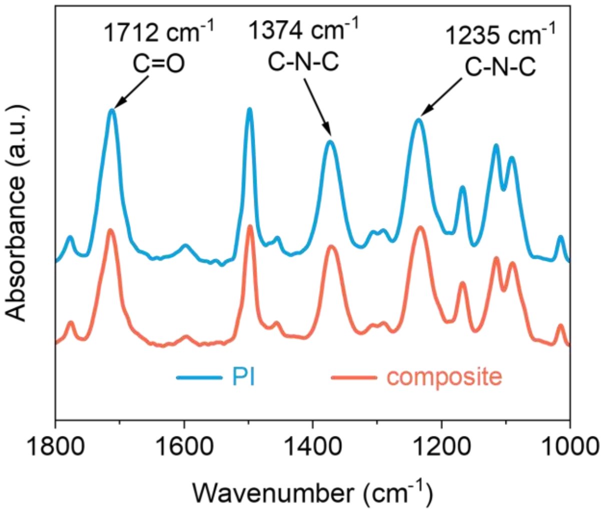

Supplementary Fig. 15 | FT-IR spectra of pure PI and composite membranes.

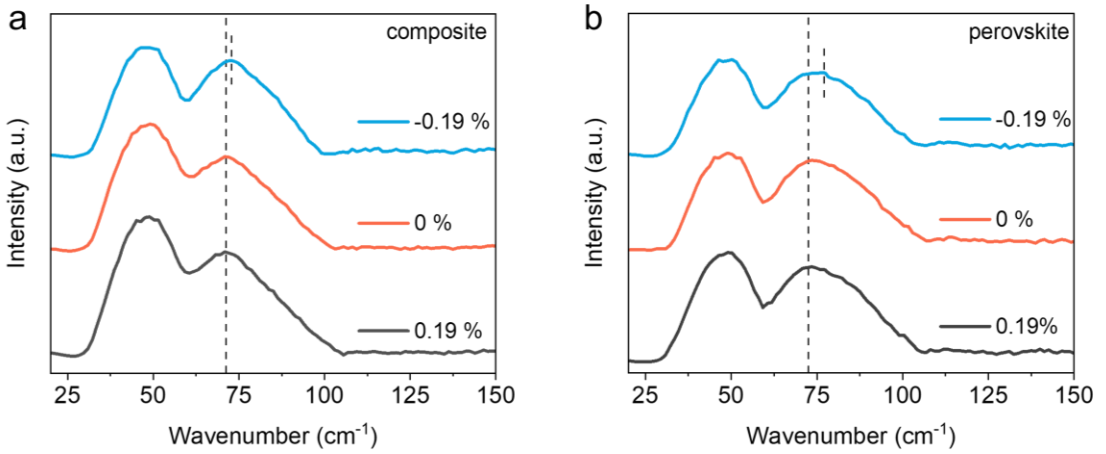

Supplementary Fig. 16 | Raman spectra of (**a**) composite membranes and (**b**) pure perovskite films under varying strain.

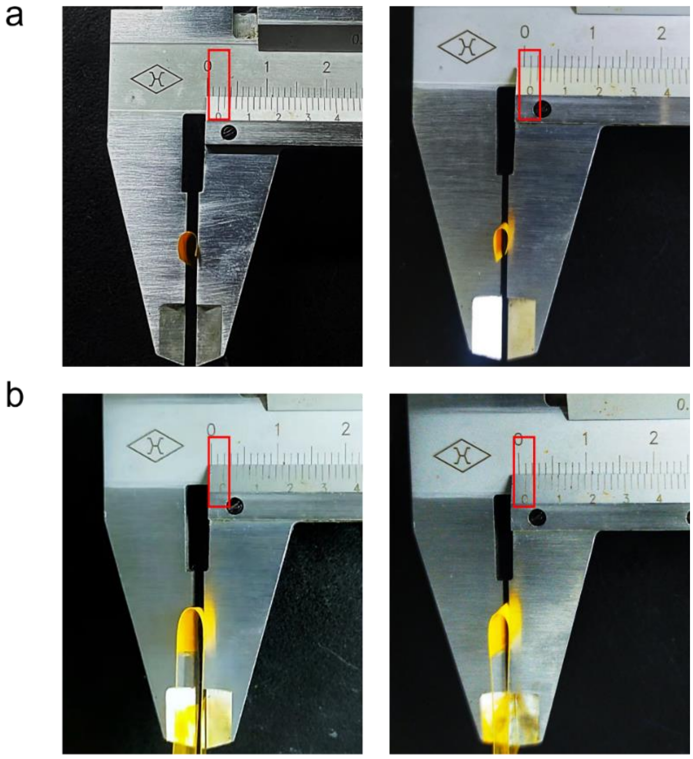

Supplementary Fig. 17 | Photographs of (**a**) free-standing composite membranes and (**b**) perovskite films on PET substrate under different bending radii.

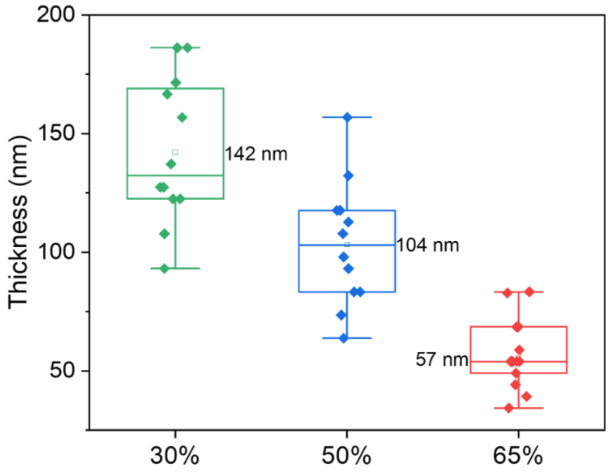

Supplementary Fig. 18 | Unit layer thickness of composite membranes with different mass fractions of perovskite.

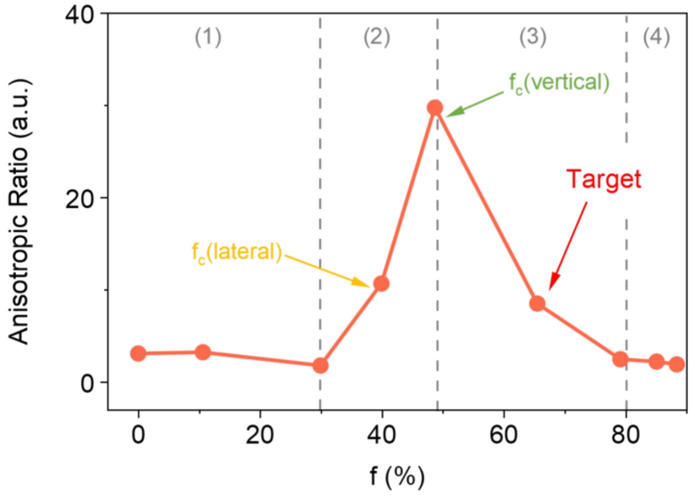

Supplementary Fig. 19 | Electrical anisotropic ratio of the composite membranes with different mass fractions of perovskite derived from the lateral and vertical resistivities. The curve can be divided into four regions: (1) When the filler fraction < 30%, the composite is non-conductive in both directions with low anisotropy ratio. (2) With filler fraction of 30% ~ 50%, the lateral direction reaches its critical threshold first, while the vertical direction remains non-conductive. (3) In the range of 50% ~ 80%, vertical conduction improves obviously, leading to the reduction of anisotropy ratio. (4) At filler fraction above 80%, the polymer phase loses its continuity and leads to low anisotropy.

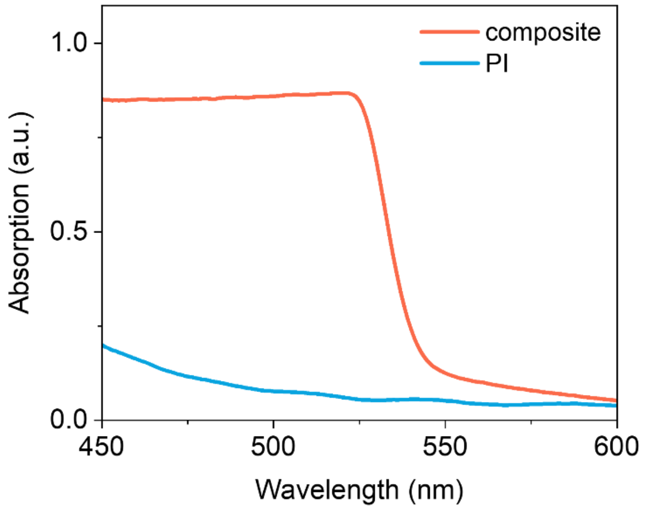

Supplementary Fig. 20 | UV-vis absorption spectra of pure polyimide and composite membranes.

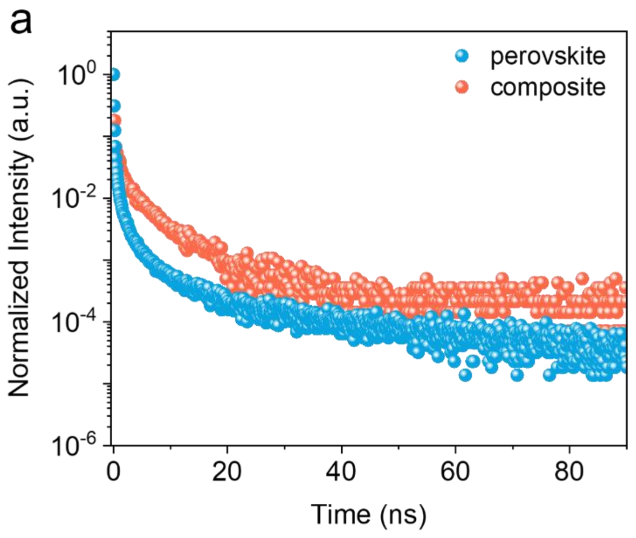

Supplementary Fig. 21 | Time-resolved PL spectra of the pure perovskite film and the composite membrane. The carrier lifetime of the composite membrane was slightly longer than that of pure perovskite film.

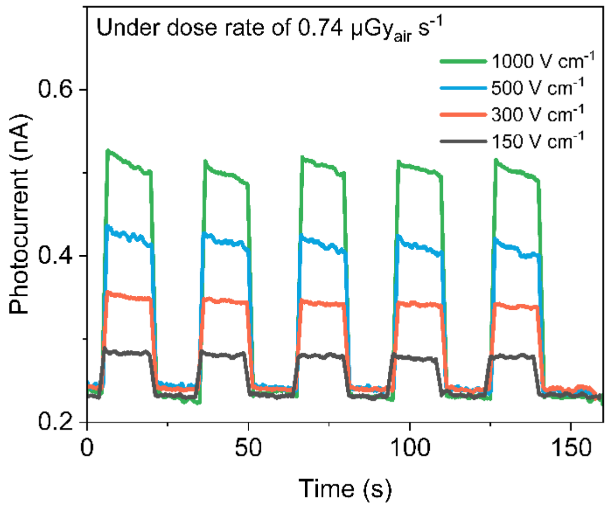

Supplementary Fig. 22 | Response current of the composite detector to X-ray pulses under varying applied bias voltage. The X-ray dose rate is 0.74 μGy_{air} s^{-1}.

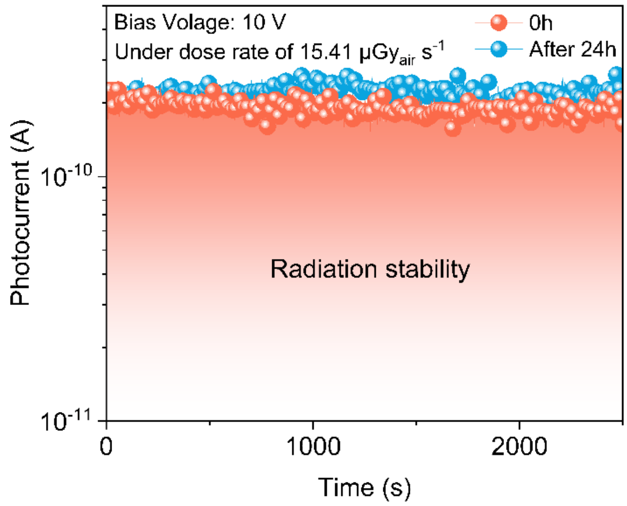

Supplementary Fig. 23 | Radiation stability of the composite detector. The X-ray dose rate is 15.41 μGy_{air} s^{-1} and the working voltage is 10 V. The detector remained radiometrically stable after storage in air for 24 h.

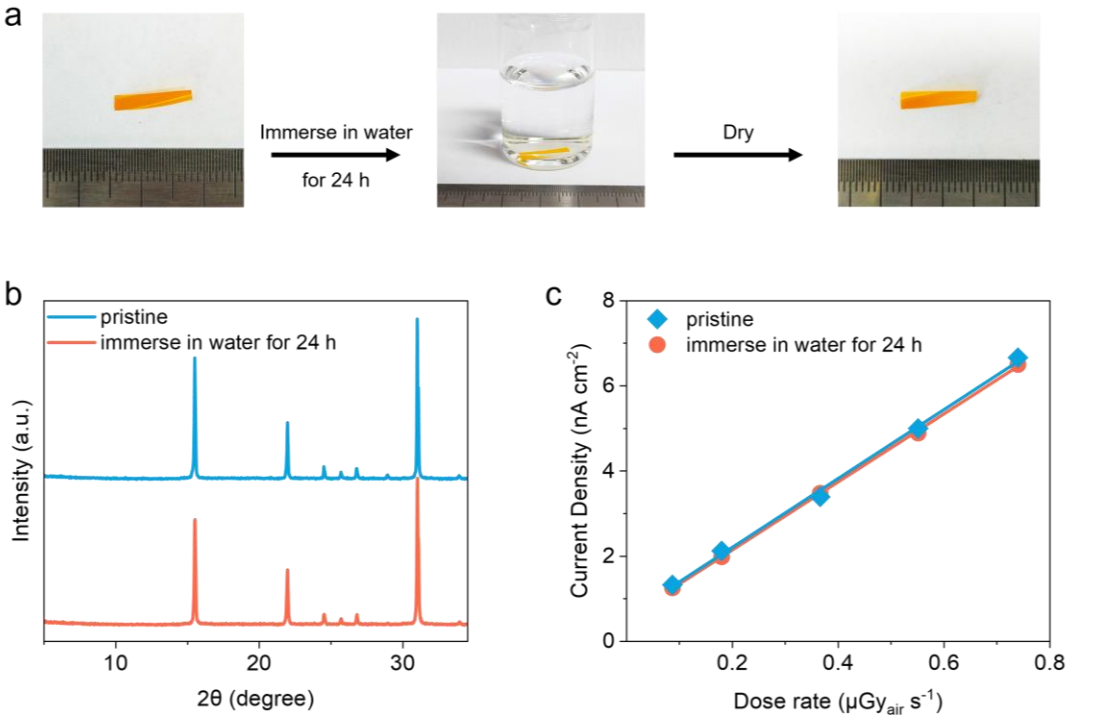

Supplementary Fig. 24 | Water stability of composite membranes. (**a**) Photographs, and (**b**) XRD patterns of composite membranes before and after immersion in water for 24 h. **c**, Current density as a function of incident dose rate of the X-ray detectors.

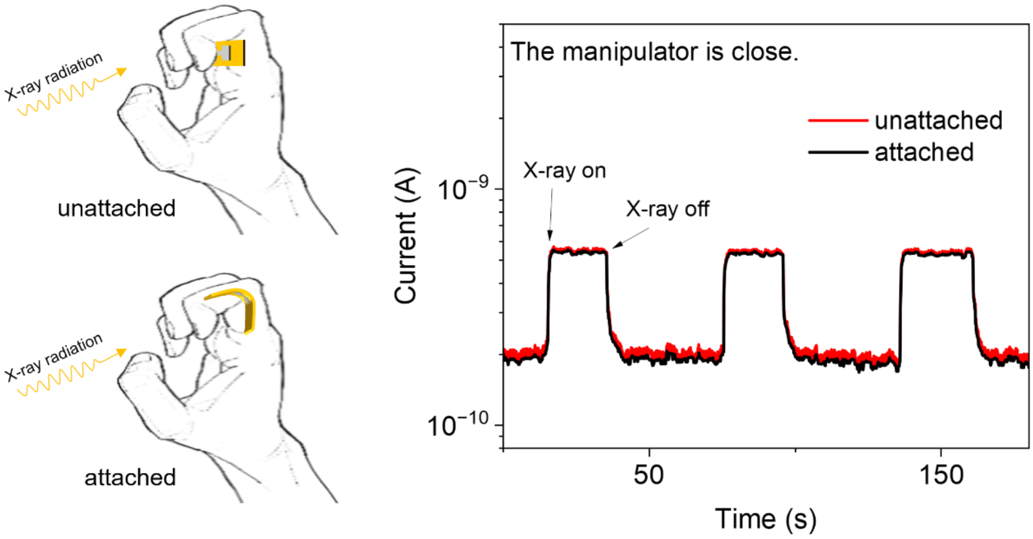

Supplementary Fig. 25 | X-ray response current of a composite detector when attached or unattached on the manipulator. Schematic is on the left. The response currents were similar whether the detectors were attached to a clenching manipulator to keep bending (0.54 nA) or unattached to release strain (0.55 nA).

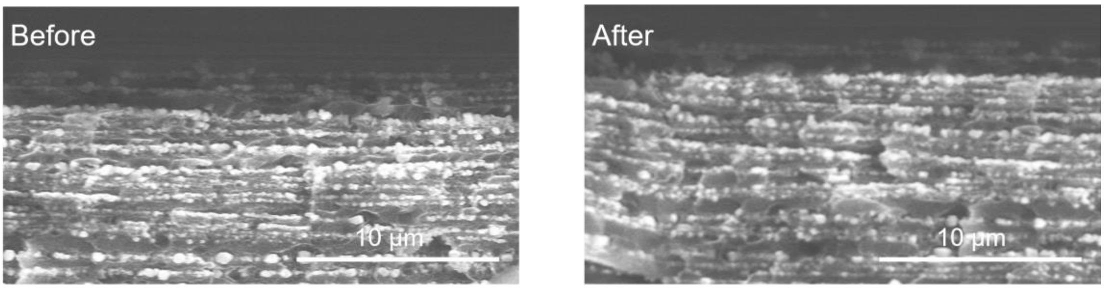

Supplementary Fig. 26 | Cross-sectional SEM images of composite membranes before and after 10,000 times bending at a bending radius of 1.5 mm. Scale bars are 10 μm.

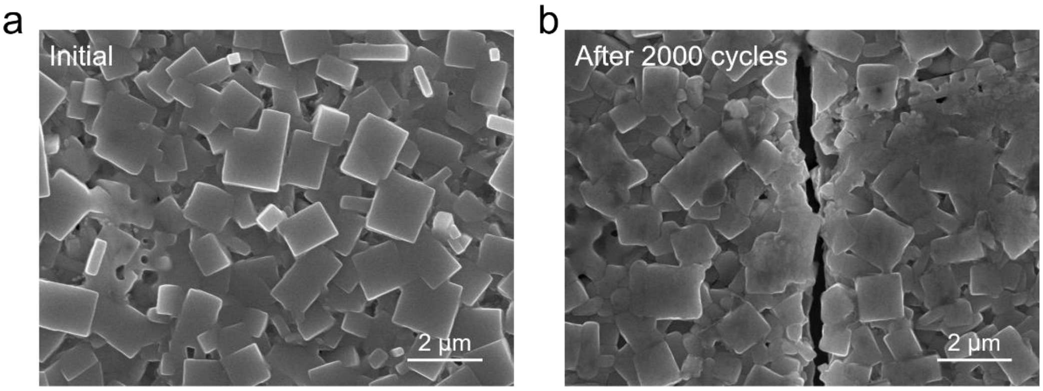

Supplementary Fig. 27 | Top-view SEM images of the perovskite/PET film (**a**) before and (**b**) after 2000 bending cycles at a bending radius of 1.5 mm. An obvious crack appeared on the surface of perovskite film. Scale bars are 2 μm.

Supplementary Table 1 | A summary of the mechanical tolerance of reported flexible devices.

Flexibility type	Device type	Bending radius /angle	Bending cycles	Retention of performance	Year	Ref
**Flexible array**	X-ray detector	n.a. (120°)	200	88%	2019	[1]
	solar cell	5 mm	300	93.7%	2020	[2]
**Flexible substrate**	photodetector	7 mm	1,000	90%	2016	[3]
	solar cell	2 mm	5,000	85%	2017	[4]
	solar cell	5 mm	10,000	90%	2021	[5]
	solar cell	5 mm	5,000	93%	2023	[6]
	solar cell	3 mm	15,000	97%	2023	[7]
	solar cell	5 mm	2,000	98.4%	2023	[8]
	solar cell	5 mm	20,000	92%	2024	[9]
	solar cell	2 mm	5,000	84%	2024	[10]
	solar cell	7 mm	10,000	94%	2024	[11]
	laser	3.5 mm	10,000	95%	2023	[12]
**Free-standing**	X-ray detector	n.a. (180°)	3,000	96%	2022	[13]
	X-ray detector	n.a. (120°)	1,000	87%	2022	[14]
	X-ray detector	2.5 mm	500	70%	2024	[15]
	X-ray detector	1.5 mm	30,000	97.4%	2025	This work

Supplementary Note 1

Calculation of the bending strain.

The applied global bending strain (**ε**') can be measured according to the following relationship:

$$\boldsymbol{\varepsilon}' = \frac{h/2}{\mathbf{R}} \tag{1}$$

where h was 0.05 mm as the thickness of PET substrate, and **R** was bending curvature. The corresponding strains from tensile to compressive on PET were 0.19%, 0% and -0.19%, respectively.

Supplementary Note 2

Calculation of the strain from XRD.

The strains (ε) under different bending strains (**ε**') can be calculated from the shift of XRD peaks:

$$\varepsilon = \frac{d_{strained(100)} - d_{non-strained(100)}}{d_{non-strained(100)}} \tag{2}$$

where $d_{strained(100)}$ was the plane spacing of films based on PET substrates and $d_{non-strained(100)}$ was the crystal plane spacing of $CsPbBr_3$ crystal without strain.

Supplementary Note 3

Finite element simulations method.

A quasi-static finite element simulation was applied to the nanoscale heterophase (layer-by-layer) structure model of PI/perovskite nanocomposite membrane that consisted of four PI membranes and three perovskite membranes. The length and thickness of PI monolayer were set to be 12 μm and 100 nm, respectively. The perovskite membranes were sandwiched between PI layers, and were composed of nanoparticles with radius of 50 nm. The spaces between perovskite nanoparticles were

filled by PI. All materials in this study were assumed to be isotropic and homogenous for simplicity. Their interfaces were integrated as indicated by the real situation of the integrated membranes. Young's modulus of the PI layers was assigned to be 4 GPa, while that of the perovskite nanoparticles was 20 GPa, respectively. Their Poisson's ratios were set to be 0.35 and 0.33, respectively. The simulation included non-linear geometric effects to enable finite deformation. The out of plane deformation was achieved by moving two bottom surface edge points inwards along x-axis, while the finite deformation was enabled by considering non-linear geometric effects. A bending angle of 50° would be reached for the curved model under steady-state, which was close to the experiment data.

The reference model employed 700 nm thick perovskite membrane and 500 nm thick PET film, the latter of which was hidden from view. The length and bending angle were set to be as same as the above model. The Young's modulus and Poisson's ratio remained the same as the above model for perovskite membrane, but set to be 2.8 GPa and 0.33 for PET film.

Supplementary Note 4

Critical resistivity behavior in composite devices near percolation.

Continuously adding perovskite with low resistivity (ρ_1) to PI with high resistivity (ρ_2), a drop in the value of the composite membrane resistivity (ρ) occurred when the mass fraction f of the perovskite approached the critical threshold fc, i.e., a percolation behavior. When f just exceeded fc, the conductive perovskite formed a continuous percolating path throughout PI. Thus, ρ was mainly determined by ρ_1 as the following relationship:

$$\rho \propto \frac{\rho_1}{(f-f_c)^t} \tag{3}$$

where t was the critical exponent calculating from the best-fit line of the plot of log (ρ) versus log (f-f_c).

Supplementary References

1. Liu, J. *et al.* Flexible, printable soft-X-ray detectors based on all-inorganic perovskite quantum dots. *Adv. Mater.* **31**, 1901644 (2019).
2. Lei, Y. *et al.* A fabrication process for flexible single-crystal perovskite devices. *Nature* **583**, 790–795 (2020).
3. Chen, S. *et al.* A flexible UV-vis-nir photodetector based on a perovskite/conjugated-polymer composite. *Adv. Mater.* **28**, 5969-5974 (2016).
4. Yoon, J. *et al.* Superflexible, high-efficiency perovskite solar cells utilizing graphene electrodes: towards future foldable power sources. *Energy Environ. Sci.* **10**, 337-345 (2017).
5. Dong, Q. *et al.* Flexible perovskite solar cells with simultaneously improved efficiency, operational stability, and mechanical reliability. *Joule* **5**, 1587-1601 (2021).
6. Wu, Y. *et al.* In situ crosslinking-assisted perovskite grain growth for mechanically robust flexible perovskite solar cells with 23.4% efficiency. *Joule* **7**, 398-415 (2023).
7. Liu, H. *et al.* A 0D additive for flexible all-inorganic perovskite solar cells to go beyond 60 000 flexible cycles. *Adv. Mater.* **35**, 2300302 (2023).
8. Min, J. *et al.* An autonomous wearable biosensor powered by a perovskite solar cell. *Nat. Electron.* **6**, 630–641 (2023).
9. Wu, Y. *et al.* Stereoscopic polymer network for developing mechanically robust flexible perovskite solar cells with an efficiency approaching 25%. *Adv. Mater.* **36**, 2403531 (2024).
10. Xu, W. *et al.* Multifunctional entinostat enhances the mechanical robustness and efficiency of flexible perovskite solar cells and minimodules. *Nat. Photon.* **18**, 379–387 (2024).
11. Tang, L. *et al.* All-round passivation strategy yield flexible perovskite/$CuInGaSe_2$ tandem solar cells with efficiency exceeding 26.5%. *Adv. Mater.* **36**, 2402480 (2024).

12. Cao, X. *et al.* Low-threshold, external-cavity-free flexible perovskite lasers. *Adv. Funct. Mater.* **33**, 2211841 (2023).

13. Wang, S. *et al.* Vertically oriented porous PET as template to integrated metal halide for high-performance large-area and ultra-flexible X-ray detector. *Small* **18**, 2205095 (2022).

14. Cui, Q. *et al.* Efficient eco-friendly flexible X-ray detectors based on molecular perovskite. *Nano Lett.* **22**, 5973-5981 (2022).

15. Li, H. *et al.* Flexible large-scale self-driven perovskite X-ray detector by precise heterogeneous integration. *ACS Energy Lett.* **9**, 64-74 (2024).
